# Regulation of enteric nervous system via sacral nerve stimulation in opioid-induced constipated rats

**DOI:** 10.3389/fnins.2023.1146883

**Published:** 2023-06-02

**Authors:** Liyun Wang, Payam Gharibani, Yi Yang, Yu Guo, Jieyun Yin

**Affiliations:** ^1^Department of Gastroenterology, The First Affiliated Hospital of Shandong First Medical University & Shandong Provincial Qianfoshan Hospital, Jinan, China; ^2^Division of Neuroimmunology, Department of Neurology, Johns Hopkins University School of Medicine, Baltimore, MD, United States; ^3^Division of Gastroenterology and Hepatology, Department of Medicine, Johns Hopkins University School of Medicine, Baltimore, MD, United States; ^4^Department of Gastroenterology, The First Affiliated Hospital of Dalian Medical University, Dalian, China

**Keywords:** sacral nerve stimulation, opioid-induced constipation, autonomic functions, enteric neurons, neuromodulation

## Abstract

**Objectives:**

Sacral nerve stimulation (SNS) has been employed for treating constipation. However, its mechanisms involving enteric nervous system (ENS) and motility are largely unknown. In this study, we investigated the possible ENS involvement of SNS in treating Loperamide-induced constipation in rats.

**Methods:**

Experiment-1 was designed to study the effects of acute SNS on whole colon transit time (CTT). In experiment-2, we induced constipation by Loperamide and then applied daily SNS or sham-SNS for 1 week. Choline acetyltransferase (ChAT), nitric oxide synthase (nNOS), and PGP9.5 in colon tissue were examined at the end of the study. Moreover, survival factors such as phosphorylated AKT (p-AKT) and Glial cell-derived neurotrophic factor (GDNF) were measures by immunohistochemistry (IHC) and western blot (WB).

**Key results:**

(1) SNS with one set of parameters shortened CTT starting at 90 min after phenol red administration (*p* < 0.05). (2) While Loperamide induced slow transit constipation with a significant reduction in fecal pellet number and feces wet weight, daily SNS for a week resolved constipation. (3) Moreover, SNS was able to shorten whole gut transit time comparing to sham-SNS (*p* = 0.01). (4) Loperamide reduced the number of PGP9.5 and ChAT positive cells, and downregulated ChAT protein expression and upregulated nNOS protein expression, whereas these detrimental effects were significantly reversed by SNS. (5) Furthermore, SNS increased expressions of both GDNF and p-AKT in colon tissue. (6) Vagal activity was reduced following Loperamide (*p* < 0.01); yet SNS normalized vagal activity.

**Conclusion:**

SNS with appropriate parameters improves opioid-induced constipation and reversed the detrimental effects of Loperamide on enteric neurons possibly via the GDNF-PI3K/Akt pathway.

**GRAPHICAL ABSTRACT:**
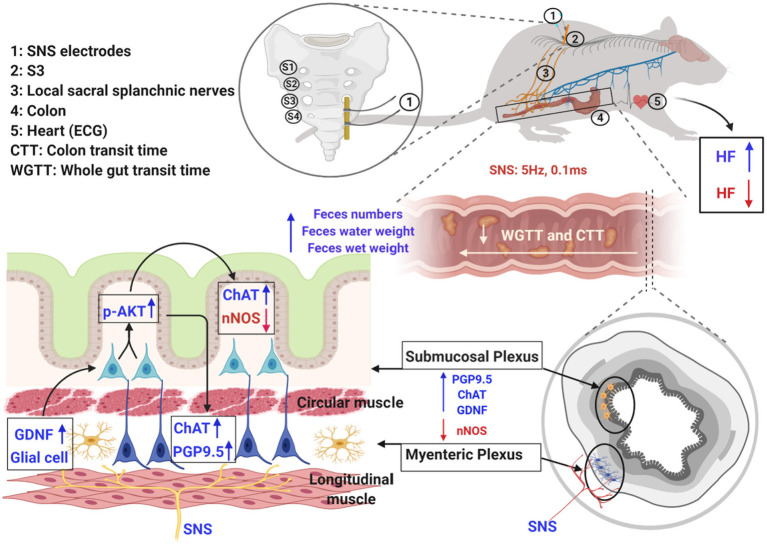

## 1. Introduction

Constipation is one of the most common digestive complaints with serious impacts on the quality of life in the United States (Camilleri et al., 2017; Peery et al., 2022), The prevalence of constipation is approximately 5–30% with a higher prevalence in female and senior (Camilleri et al., 2017). Constipation is defined as bowel movements for less than 3 times a week, with difficult or incomplete evacuation of lumpy or hard stools (Mearin et al., 2016). Constipation can cause abdominal discomfort, distension, vomiting, gut obstruction, and even fatal pulmonary embolism (Mostafa et al., 2003). With substantial overlap, several types of chronic constipation have been identified, including normal-transit constipation, slow-transit constipation (STC) and dyssynergic defecation (Camilleri et al., 2017; Black and Ford, 2018). STC is a motility disorder characterized by markedly increased bowel transit time (Wang, 2015) and accounts for a significant proportion of cases, including opioid-induced constipation (OIC; Poulsen et al., 2016). While the pathogenesis of constipation remains largely unknown, alternations in interstitial cells of Cajal (He et al., 2000), degeneration of the enteric nervous system (ENS) and abnormalities of the enteric neurotransmitters/factors are associated with STC (Tomita et al., 2002a,b; Andromanakos et al., 2015). Neuropathological changes of both cholinergic and nitrergic neurons as well as glial and nerve derived neurotrophic factors have been shown to be associated with STC (Bassotti et al., 2006; Dickson et al., 2010; Rodrigues et al., 2011). OIC is a debilitating adverse effect associated with opioids use and is present in 60–90% of opioid users (Morlion et al., 2015; Poulsen et al., 2015). The actions of opioids are mediated through G-protein coupled μ- and δ- receptors (Williams et al., 2013) which are the principal opioid receptors in GI tract; This two receptors expressed predominantly in ENS (Bagnol et al., 1997; Nelson and Camilleri, 2016) and cause membrane hyperpolarization, decrease neurotransmitter release and hence, impaired motility (Diego et al., 2011; Galligan and Akbarali, 2014). Current treatment for STC includes dietary management, physical exercise, enemas, drug treatment, biofeedback psychosocial treatments, and surgical treatment (Corsetti and Tack, 2014). However, the treatment success is still unsatisfactory (Hanson et al., 2019). Fiber treatment or stimulant laxatives often do not target the multiple symptoms associated with chronic constipation or might alleviate one symptom and exacerbate another (Johanson and Kralstein, 2007). Moreover, the treatments used for functional constipation are not able to satisfy patients with opioid-induced constipation (Hanson et al., 2019). On the other hand, surgical approaches such as subtotal colectomy and total colectomy may adversely affect the quality of life due to the risk of postoperative diarrhea and/or incontinence (Han et al., 2018).

With the emergence of new treatment approaches such as neuromodulation for treating pain and inflammatory diseases, sacral nerve stimulation (SNS) has been considered a potential feasible approach for treating other GI disorders (Parittotokkaporn et al., 2020). SNS has been approved by FDA for treating fecal incontinence and overactive bladder (Thin et al., 2013). In addition, the utilization of SNS has been expanded to several other GI dysfunctions, such as pelvic pain and irritable bowel syndrome (Falletto et al., 2009; Lundby et al., 2011). Interestingly, SNS has shown some promises in improving idiopathic constipation (Holzer et al., 2008; Naldini et al., 2010; Ortiz et al., 2012; van Wunnik et al., 2012). In one recent study of our lab, we found that SNS with appropriate parameters was effective in enhancing rectal compliance and distal colon transit (Huang et al., 2019). However, the exact mechanisms of actions of SNS on ENS remained largely unknown. The ENS known as the “little brain,” contains large amounts of neurons and regulates various GI functions, including contraction of intestine in an independent manner (Mearin et al., 2016). Several studies have shown that STC is mostly caused by disorders of the relevant nerves, especially the ENS (Tomita et al., 2002a; Nelson and Camilleri, 2016; Tu et al., 2020a).

The aims of this study were (1) to study the effect of SNS with various parameters on whole colon transit in normal rats; (2) to investigate the effect of SNS on constipation in Loperamide-induced constipated rats and (3) to explore mechanisms of SNS action involving ENS neurons and specific GDNF-PI3K/Akt pathway.

## 2. Materials and methods

### 2.1. Animals and ethic statement

Thirty-eight 10–12 week-Sprague–Dawley rats (male, 270–320 g, Charles River Laboratories, MA) were used in accordance with the guidelines of the Johns Hopkins University for the Care and Use of the laboratory Animals (ACUC’s approved protocol #RA17M296). The rats were housed in an animal room with regulated temperature (20–22°C), 50% humidity, and a 12-h light/12-h dark cycle with free access to water and solid food. Cefalexin (15 mg/kg, ip) was administered prior to surgery and every 12 h for 24 h to prevent post-operative infection. Buprenorphine sustained release (0.56 mg/kg, sc) was injected once before the surgery.

### 2.2. Surgical procedure

Rats were anesthetized with 2% isoflurane (Abbott Laboratories, IL, USA) with a 1–2 liter/min oxygen flow. Electrodes were implanted for recording electrocardiogram (ECG) for measuring heart rate variability (HRV) and stimulating sacral nerve at S3. For recording ECG, three electrodes (A&E Medical, Farmingdale, NJ) were implanted subcutaneously (two of them under the skin of the chest, one under the skin of abdomen; Huang et al., 2019; Tu et al., 2020a). For sacral nerve stimulation (SNS), a posterior midline incision was performed, and a pair of electrodes (cardiac pacing wire, A&E Medical, Farmingdale, NJ) was placed around right sacral nerve (S3) behind the sacral foramen and fixed by a surgical knot 2–3 mm apart (oval cathode 2–3 mm in length in each electrode) and the dental cement was used to isolate the exposed wires from circumjacent tissues (Huang et al., 2019; Tu et al., 2020a). Electrode connecting wires were subcutaneously tunneled through the back and externalized at the middle neck back. The skin incisions were closed with sutures and the rats were housed individually to avoid connecting wires being chewed up by other rats.

In experiment 1, 3 days after the electrode placement, the rats were anesthetized and a catheter (PE90; Becton Dickinson, United States i.d., 0.86 mm; o.d., 1.27 mm) was inserted into the ileocolonic junction and advanced to the proximal colon (1 cm distal to the ileocolonic junction; Liu and Chen, 2006). The detailed operation procedure was: The abdominal incision exposes the ileocecal intestine, a fistula was made in the proximal cecum 1 cm away from the ileocolonic junction, the catheter was inserted through the fistula into the proximal cecum, the length of the intestinal catheter is about 3 cm, the fistula and the catheter were sewn and fixed by a purse suture. Incisions were sutured in layers, the hemostat clamped the free end of the catheter, with the same way as the electrodes, tunneled subcutaneously and externalized at the middle back of the neck. Several appropriate positions of the catheter were trapped with a larger size catheter (0.5 cm) as a snap stuck inside the suture, to prevent prolapse of the catheter.

### 2.3. Experiment-1: effect of acute SNS on colon transit in normal rats

**Figure 1:**
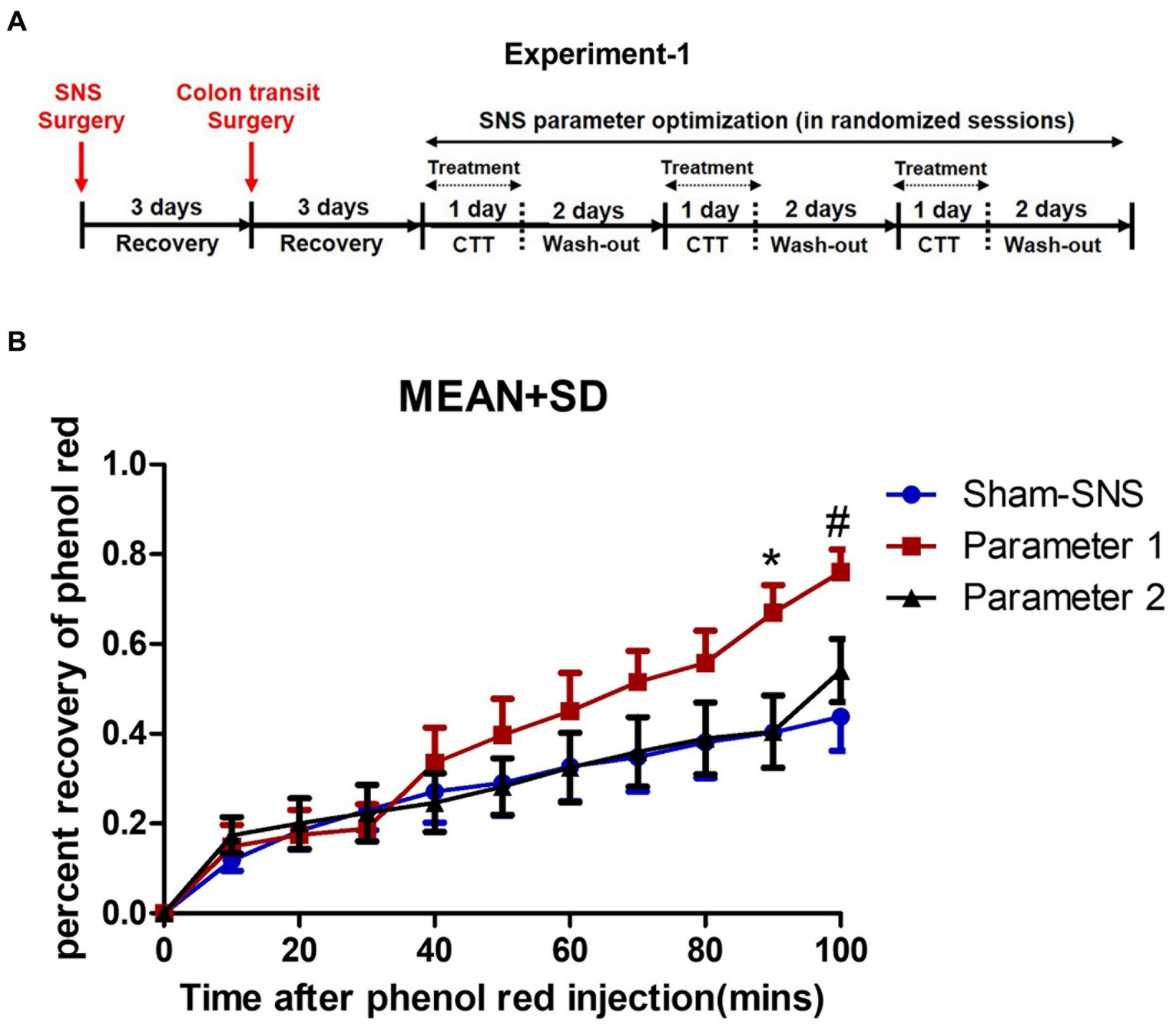
Experiment-1: effect of acute SNS with P1 (5Hz, 0.1 ms, 90% of motor threshold and free run) and P2 (5Hz, 0.5 ms, 90% of motor threshold and 10s On, 90s Off) on colon transit time of normal rats. **(A)** Study plan for acute SNS parameter optimization. **(B)** Effects of SNS with two various short pulse parameters (P1 and P2) on whole colon transit measured by percentage of recovered phenol red during 100 min after injection. To minimize the effect of session order, we randomized the sessions for SNS parameter optimization. SNS with P1 was more effective in increasing the percentage of recovered phenol red. Values were represented as the means ± SE (*n* = 14). Comparisons among sham, SNS-P1 and SNS-P2 were analyzed by repeat measures 2-way ANOVA with Tukey correction (^*^p ≤ 0.05, ^#^*p* ≤ 0.005). SNS, sacral nerve stimulation; P, parameter; CTT, colon transit test.

This experiment was designed to find the most effective parameters for accelerating colon transit in normal rats (Figure 1A). Fourteen normal rats were used to test the effects of SNS on colon transit with two sets of parameters, P1 (0.1 ms, 5 Hz, continuous-on and 90% motor threshold) that was reported to improve constipation in rats (Huang et al., 2019) and P2 (0.5 ms, 5 Hz, 10 s on/90 s off and 90% motor threshold: 90% of the minimum stimulation intensity) that can produce a muscle contraction; see Liu and Chen (2006) that was found to improve colonic inflammation (Guo et al., 2019; Tu et al., 2020a,b).

There was a total of three randomized sessions, including a control session (sham-SNS without stimulation) on separate days with an interval of 3 days or more. To minimize the effect of session order, we randomized the sessions for SNS parameter optimization, and each rat served as its own control to minimize the interindividual variation (Figure 1A). Rats were placed in a restrainer regardless of treatment scheme of SNS or sham-SNS. The SNS electrode wires (tunneled from the back of the neck) were connected to a universal Pulse Generator (model DS8000; World Precision Instruments, Sarasota, FL).

Rats were fasted prior to this experiment. While rats were in a restrainer, 1.5 mL saline containing phenol red (0.5 mg/mL) at the speed of 0.1 mL/min was injected into the proximal colon via the inserted catheter and the anal effluent was collected in a plastic weighting boat every 10 min for a period of 100 min for evaluation of the output percent of phenol red representing colon transit. SNS was performed via the pair of electrodes during the first 40 min of the transit test. The content of each sample was placed in a cup filled with 100 mL of 0.1 N NaOH. The mixture was maintained at room temperature for 1 h. 5 mL of the supernatant was added to 0.5 mL of trichloroacetic acid solution (20%, w/v) to precipitate the proteins. After centrifugation (2,800 rpm) for 20 min, 3 mL of the supernatant was mixed with 4 mL of 0.5 N NaOH. Optical density (OD) reflecting the absorbance of the sample was read at the wavelength of 560 nm. A standard curve was obtained by testing the OD of phenol red with gradient concentration, while blank control was set with 0.1 N NaOH solution. The concentration (C) of the solution read at 560 nm (C=OD) was determined according to the linear coefficient of the standard curve, and finally the amount of phenol red (m) recovered from each 10 min output was calculated as percent of (m = C × volume).

### 2.4. Experiment-2: effects of optimized SNS on constipation

**Figure 2:**
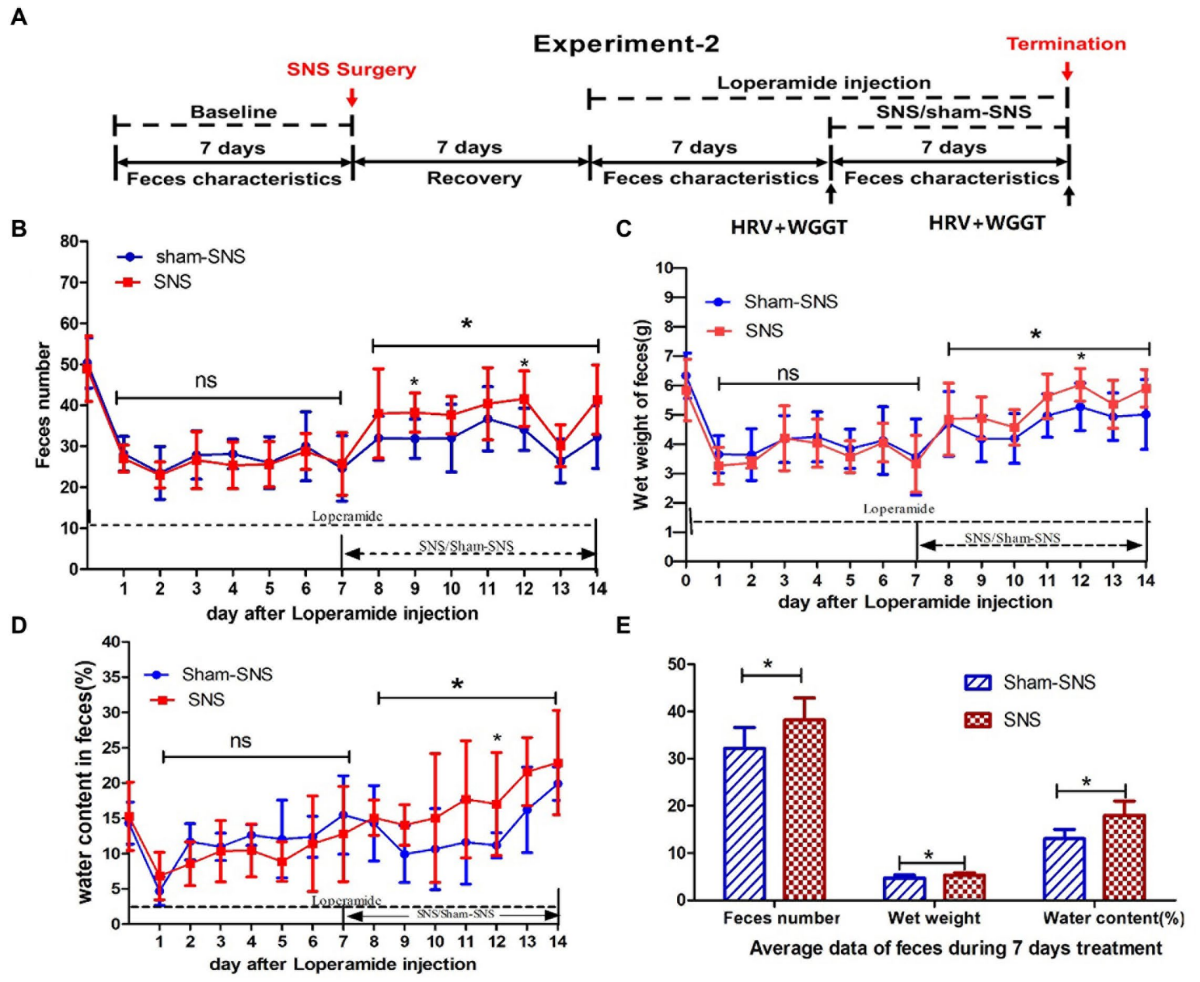
Experiment 2: effect of chronic SNS for 1 week on feces content and gastrointestinal motility in rats with Loperamide-induced constipation. **(A)** Depicting the animal study design for chronic SNS on Loperamide-induced constipated rats. After SNS with optimized parameter (P1: 5 Hz, 0.1 ms, 90% of motor threshold, free run), the number of feces significantly increased especially at the day 7 and day 12 (2 and 5 days after SNS treatment compared with sham-SNS) **(B)**, the improvement of wet weight **(C)** and the water content of feces **(D)** significantly also happened on day 12 (5 days after SNS treatment compared with sham-SNS). In this experiment, some special treatments were performed before testing the WGTT, such as fasting and oral gavage with phenol red, which may affect the characteristic of next day’s feces. Thus the improvement of feces number, wet weight and water content of feces did not happen daily during the SNS treatment, yet the average of daily data during the 7 days was significantly improved compared with sham-SNS **(E)**. Comparisons between sham and SNS groups in the number of feces, wet weight and water content were performed with repeat measures 2-way ANOVA; Comparisons between the two groups in the average index during the 7 days treatment were analyzed by unpaired *t*-test (*p ≤ 0.05, ns, not significant). SNS, sacral nerve stimulation; WGTT, whole gut transit time.

To investigate if and how the optimized SNS would improve constipation, we induced constipation in 16 rats and assessed feces characterizations as well as WGTT representing the GI motility (Figure 2A).

Rats were randomly divided into SNS and sham-SNS groups (*n* = 8) and Loperamide hydrochloride (Sigma-Aldrich, St. Louis, MO, United States) at a dose of 2 mg/kg was injected subcutaneously twice per day for 7 days (9:00 a.m., 20:00 p.m.; Huang et al., 2019). Seven days later, SNS with optimized parameter (P1) was applied for 4 h per day for 1 week while the animals were still being treated with Loperamide. Rats were housed individually in their cage on a grid cast to allow us to collect the feces and to avoid coprophagy. The ECG and WGTT were assessed before and after the treatment (Figure 2A).

Feces were collected every 24 h. While the pellet numbers were counted, wet weight and water content were measured. The water content of feces was calculated as: water content (%) = [(pellets wet weight - pellets dry weight)/pellets wet weight] × 100%. Pellet dry weight was determined as the weight after exposing in air (30% humidity) for 48 h. Feces characteristics were compared to the baseline and between treatment groups.

As shown in Figure 2A, WGTT was performed before and after the treatment. The WGTT was determined by the first appearance of phenol red in the stool, starting from the intragastric gavage of 3 mL of 0.5% Phenol red in saline.

### 2.5. Mechanisms of SNS on constipation

#### 2.5.1. Assessment of autonomic function

Vagal and sympathetic activities were assessed by spectral analysis of the heart rate variability (HRV) signal derived from the original ECG recording using a custom-made software and a previously established method (Tu et al., 2020a; Ye et al., 2020). Briefly, we used a special software developed and validated in our laboratory to identify R waves from the ECG and calculate R-R interval. The spectral analysis method was then performed on the HRV signal. The power in the low frequency band (LF: 0.3–0.8 Hz) represents mainly sympathetic activity and the power in the high frequency band (HF: 0.8–4.0 Hz) stands purely for parasympathetic/vagal activity. The LF/HF ratio reflects the balance between sympathetic activity and vagal activity. As shown in Figure 2A, HRV was measured before and after the treatment by recording ECG.

#### 2.5.2. Assessment of enteric nervous system functions

At the end of the study (Day14), rats (total *n* = 24) were euthanized by overdose of isoflurane (adjusting the isoflurane flow rate to 5% until breathing stopped) and then opening the abdominal cavity and chest for tissue collection. Middle 1/3 of colon was harvested for assessing key neurons for motility (PGP9.5, ChAT and nNOS) as well as survival growth factors by immunohistochemistry (IHC) and western blot (WB) (*n* = 8). Eight healthy rats were used as a normal control.

##### 2.5.2.1. Immunohistochemistry analysis

The colon samples were immediately fixed with 4% paraformaldehyde for 24 h upon separation, followed by paraffin embedding and cutting to a thickness of 4 μm. Sections were deparaffinized in xylene and hydrated in ethanol solutions with gradient concentration. Antigen retrieval was performed in citrate buffer (pH 6.0) for PGP9.5 and nNOS and Tris-EDTA buffer (pH 9.0) for ChAT. Inactivation of endogenous peroxidase activity was performed with 3% hydrogen peroxide (H_2_O_2_) for 10 min, then nonspecific binding was blocked by treatment with normal horse serum for 1 h at room temperature. Sections were incubated for the primary antibodies: nNOS (1:100; Cell Signaling, Boston, MA), ChAT and PGP9.5 (1:200; Abcam, Cambridge, UK) overnight at 4°C. After washing (3×) in 0.01 M PBS (pH 7.2), the slides were incubated with secondary antibody (horse anti-mouse/rabbit IgG) for 30 min at 37°C. After washing in PBS (3x), the sections were incubated with SABC complex reagent for 30 min in room temperature, then the localization of target proteins was visualized by incubating the sections for in freshly prepared 3,3-diaminobenzidine solution, the incubating time was ended by positive staining appearance under microscopic observation. The slides were washed with ddH_2_O, followed by counterstaining with hematoxylin, and finally dehydrated. Blank controls were set by the absence of primary antibody, which was replaced by PBS.

Positive immunostained cells were counted per five randomly visual fields in muscularis propria at 400× (Olympus FV500 optical microscope, Tokyo, Japan) by two observer who were blind to the study and presented as percentage of total area according to established methods.

##### 2.5.2.2. Western blot analysis

Colon tissue samples were lysed in a RIPA buffer containing a 2% phosphatase-inhibitor (Thermo-Scientific, Waltham, MA) and a 1% mammalian-protease inhibitor (Sigma-Aldrich).

50 μg of extracted proteins were run a single track of 10% sodium dodecyl sulfate–polyacrylamide gel electrophoresis (SDS–PAGE), and the separated proteins were transferred electrophoretically onto cellulose membranes. The membranes were blocked in 5% non-fat dry milk for 60 min, and then incubated with primary antibodies against nNOS and p-Akt (both 1:1,000; Cell Signaling, Boston, MA); ChAT (1:100), GDNF (1:500) and GAPDH (1:10,000) (Abcam, Cambridge, UK) overnight at 4°C. The membranes were washed 3x with TBS-T (TBS mixed with 0.1% Tween-20) and then, they were incubated with ECL AP-conjugated anti-rabbit/mouse IgG (1:3,000; GE Healthcare, UK) for 90 min in room temperature. Quantitative analysis was done using Image-J software (NIH, Bethesda, MD).

### 2.6. Statistical analysis

All results are expressed as mean ± SE. Shapiro–Wilk test was employed for testing normal distribution of data. Paired or unpaired *t*-test was used to compare the difference between two groups. Differences between multiple groups were evaluated by repeat measures one-way or two-way ANOVA with *post-hoc* Tukey test. *p* value ≤ 0.05 was significant. All statistical analysis was performed by observers who were blind to the study using SPSS 26.0 statistics package (SPSS Inc., Chicago, IL) and Graph Pad Prism 9.0 version (Graph Pad, San Diego, CA).

## 3. Results

### 3.1. Effects of SNS on whole colon transit

As shown in Figure 1B SNS with parameter 1 (previously found effective in improving constipation) but not parameter 2 was able to enhance the colon transit compared to sham-SNS: there was a gradual increase in colon transit (or emptying) with SNS of P1 and this increase became significant at 90 min and 100 min compared with other groups. At 90 min, the percentage of phenol red recovered from the anus increased from 40.3 ± 29.5% in the control session (sham-SNS) to 66.9 ± 19.6% with SNS (P1), about 66% increase in transit. Meanwhile, at 100 min, the percentage of phenol red recovered from the anus increased from 43.8 ± 28.3% in the control session (sham-SNS) to 76.0 ± 16% with SNS (P1), about 73.5% increase in transit. In contrast, SNS with parameter-2 (previously found effective in suppressing intestinal inflammation) was not able to alter colon transit compared to sham-SNS.

### 3.2. Effects of optimized SNS-P1 on constipation

As shown in Figure 2B, 7-day Loperamide administration significantly reduce the number of fecal pellets by ~50% (both *p* < 0.001) in all groups compared to their corresponding baseline: sham-SNS (50.36 ± 6.12 in baseline vs. 20.71 ± 6.40 in day7 of Loperamide) and SNS (48.93 ± 7.98 in baseline vs. 25.38 ± 5.32 in day7 of Loperamide); both *p* < 0.002. We found that SNS treatment for 7 days increased the number of fecal pellets compared to sham-SNS, especially 2 days (38.3 ± 4.77 vs. 31.86 ± 4.78, *p* < 0.05), 5 days (41.63 ± 6.76 vs. 34.14 ± 5.15, *p* < 0.05) after SNS treatment initiation, although the increasements were not significant on some single days after SNS treatment, the average of the feces number during the 7 days for SNS was significantly increased than that of the Sham-SNS group (38.20 ± 4.69 vs. 32.2 ± 4.44, *p* < 0.05; Figure 2E), Similar results were observed in the wet weight of fecal pellets (Figure 2C). After 7 days of Loperamide administration, the wet weight of feces decreased in SNS from 5.84 ± 1.05 gr. in baseline to 3.33 ± 0.97 gr. and in sham-SNS from 6.34 ± 0.78 gr. in baseline to 3.56 ± 1.3 gr, both *p* < 0.005. Similarly, SNS treatment significantly increased the wet weight of fecal pellets compared to sham-SNS, especially on 5 days after treatment (6.02 ± 0.56 vs. 5.27 ± 0.68, *p* < 0.05), and the average wet weight of fecal pellets during 7 days of SNS treatment was also significantly increased than Sham-SNS (5.33 ± 0.5 gr vs. 4.74 ± 0.6 gr, *p* < 0.05; Figure 2E). Similarly, SNS treatment for was also able to increase the percentage of water content of fecal pellets compared to sham-SNS, especially 5 days after treatment (0.17 ± 0.07 vs. 0.11 ± 0.06, *p* < 0.05), also the average of this index during 7 days of SNS treatment was also significantly increased compared with Sham-SNS (0.18 ± 0.03 vs. 0.13 ± 0.02, *p* < 0.05; Figures 2D,E).

### 3.3. Effects of SNS-P1 on gastrointestinal transit

**Figure 3:**
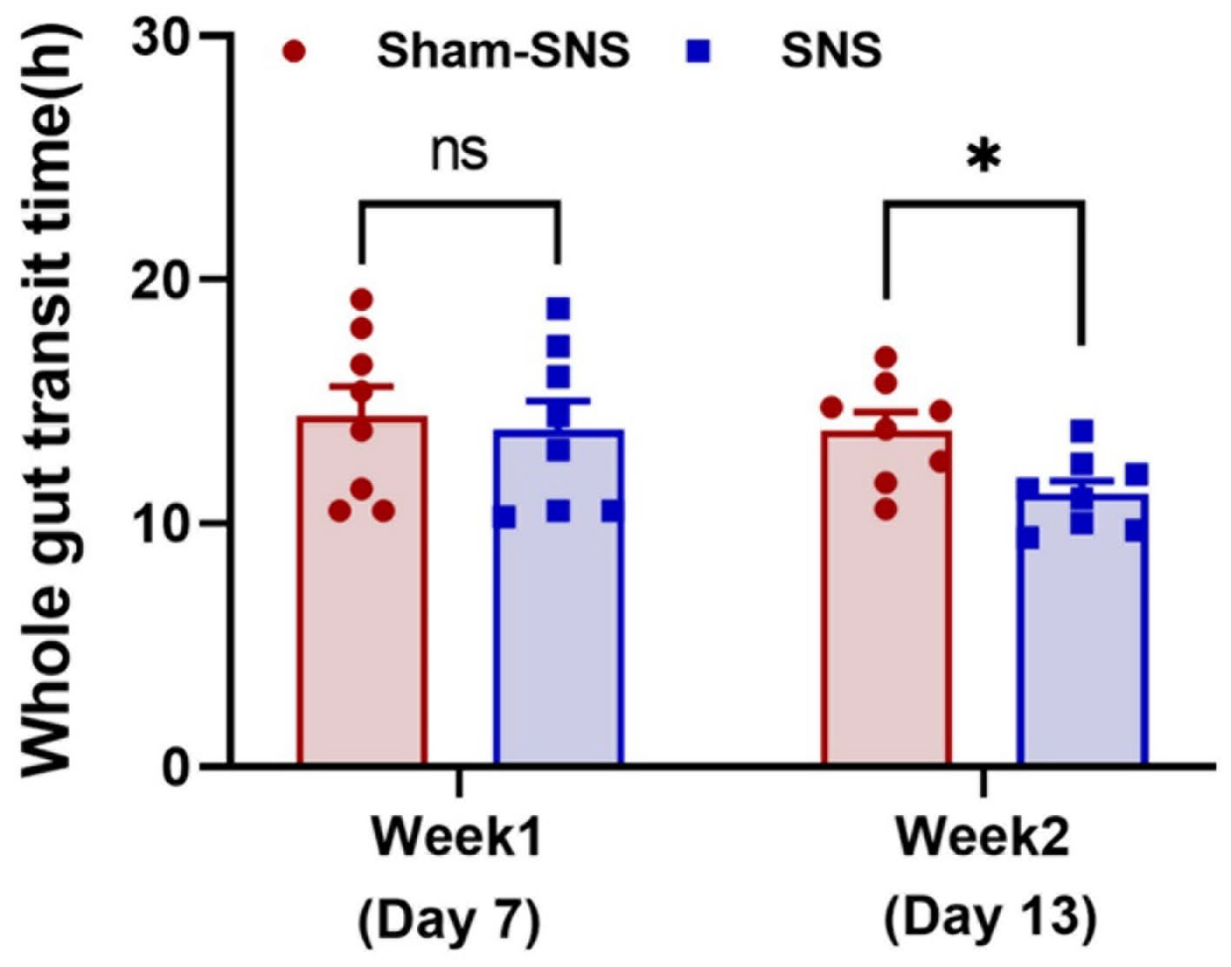
Showing WGTT at the end of week 1 (animal model) and week 2 or the day 13 (SNS/sham-SNS treatment) measured by appearance of phenol red given orally. SNS but not sham-SNS (no stimulation) for 4 h/day for 7 days significantly reduced WGTT after 1-week treatment. Values were represented as the means ± SE (*n* = 8). Comparisons between the two groups in WGTT were analyzed by unpaired *t*-test (*p ≤ 0.05, ns, not significant). WGTT, whole gut transit time; HRV, heart rate variability; NS, not significant.

WGTT was carried out before treatment initiation (1-week after Loperamide administration) as well as 6 days after treatment (day 13). As shown in Figure 3 after 1-week Loperamide administration, orally-gavaged phenol red was emerged after ~13 h in both randomly divided sham-SNS and SNS groups. However, 6 days SNS treatment significantly shortened the WGTT by 23.17% (*p* = 0.01) compared to sham-SNS group (11.22 ± 0.91 h vs. 13.82 ± 2.6 h, respectively).

## 4. Mechanism of actions of SNS for constipation

### 4.1. Autonomic functions

**Figure 4:**
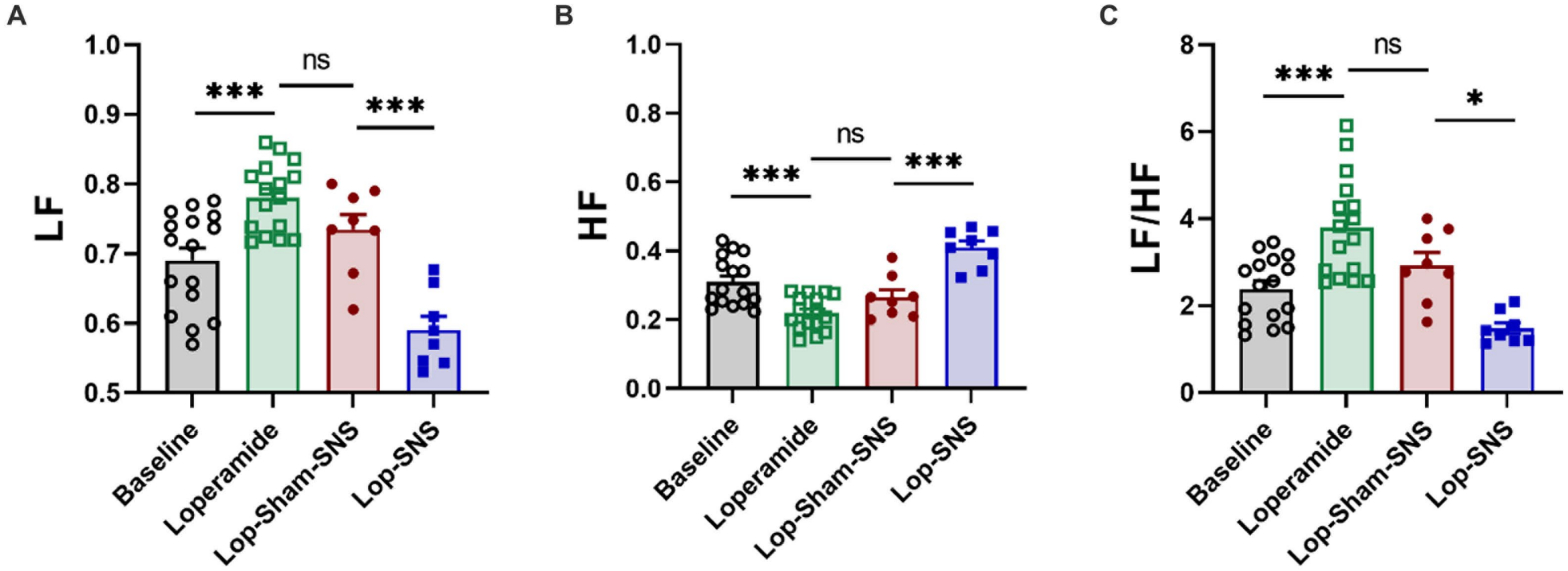
Effect of chronic SNS for 1 week on autonomic function presenting by vagal and sympathetic activities in Loperamide-induced constipated rats. LF and HF were measured by heart rate variability derived from ECG recording. LF (0.3–0.8 Hz) represents sympathetic activity, while HF (0.8–4.0 Hz) represents vagal activity. LF/HF ratio reflects the balance between sympathetic and vagal activity. Chronic SNS (but not sham-SNS) for 4 h/day for 7 days, significantly increased vagal activity (HF; **B**) and decreased sympathetic activity (LF; **A**) and the balance between sympathetic and parasympathetic activity (LF/HF; **C**) after 1-week treatment. Values were represented as the means ± SE (*n* = 8). Comparisons between the baseline and Loperamide were performed with paired *t*-test. Meanwhile comparisons among Loperamide, sham-SNS and SNS groups were performed with One-way ANOVA with Tukey correction (*p ≤ 0.05, ***p ≤ 0.0005, ns, not significant). SNS, sacral nerve stimulation; HRV, heart rate variability; HF, high frequency; LF, low frequency.

As shown in Figure 4, following 1-week treatment, SNS significantly decrease sympathetic activity (LF) (0.59 ± 0.01 vs. 0.73 ± 0.02; *p* < 0.001) and increased vagal activity (HF) (0.73 ± 0.02 vs. 0.59 ± 0.01; *p* < 0.001) compared to sham-SNS (Figures 4A,B, respectively). Sympathovagal balance (Figure 4C) showed a markedly increase in value following 1-week Loperamide administration (Loperamide: 3.8 ± 0.0.3 vs. baseline: 2.38 ± 0.18, *p* < 0.01), whereas SNS demonstrated to reverse the effects of Loperamide significantly comparing to sham-SNS (SNS: 1.48 ± 0.12 vs. sham-SNS: 2.93 ± 0.29, *p* = 0.01).

### 4.2. Enteric nervous system functions

**Figure 5:**
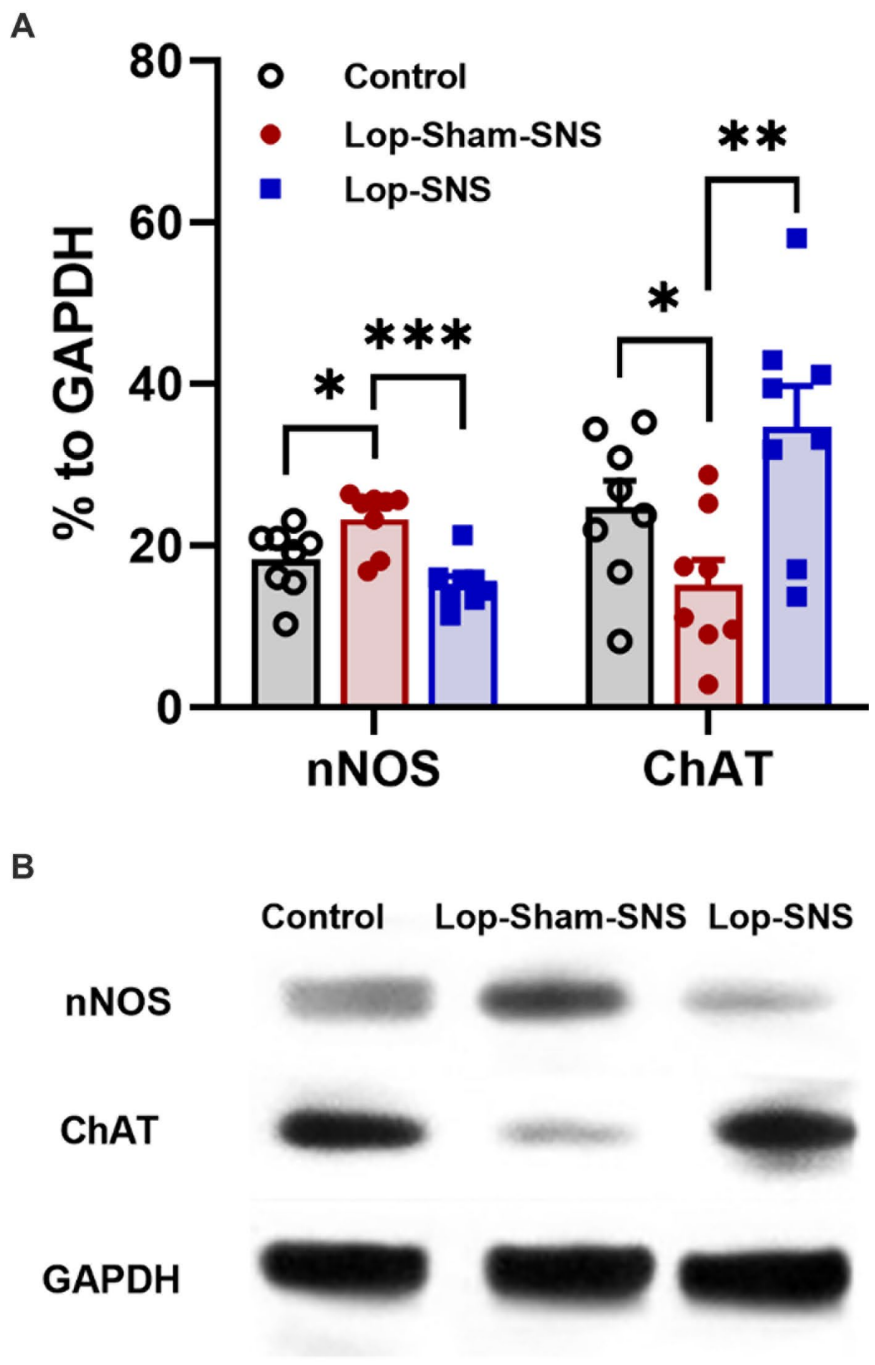
Effect of chronic SNS on protein expression of enteric neurons of ChAT and nNOS in Loperamide-induced constipated rats in comparison with control (normal) rats **(A)**. This graph shows the analyzed protein expression of ChAT and nNOS in colon tissue (middle third) in control, untreated (sham-SNS) and treated (SNS) groups. SNS markedly upregulated ChAT protein expression in colon tissue after 7 days. In contrast, nNOS that was upregulated in sham-SNS group was normalized by SNS. **(B)** Shows the protein expression of nNOS (170 kDa) and ChAT (72 kDa) and GAPDH (36 kDa) in colon tissue measured by Western-blot. Values were represented as the means ± SE (*n* = 8) as percentage of relative intensity to internal control (GAPDH). Comparisons between control and sham-SNS as well as between sham-SNS and SNS were conducted with unpaired *t*-tests. SNS, sacral nerve stimulation (*p ≤ 0.05, **p ≤ 0.005, ***p ≤ 0.0005).

**Figure 6:**
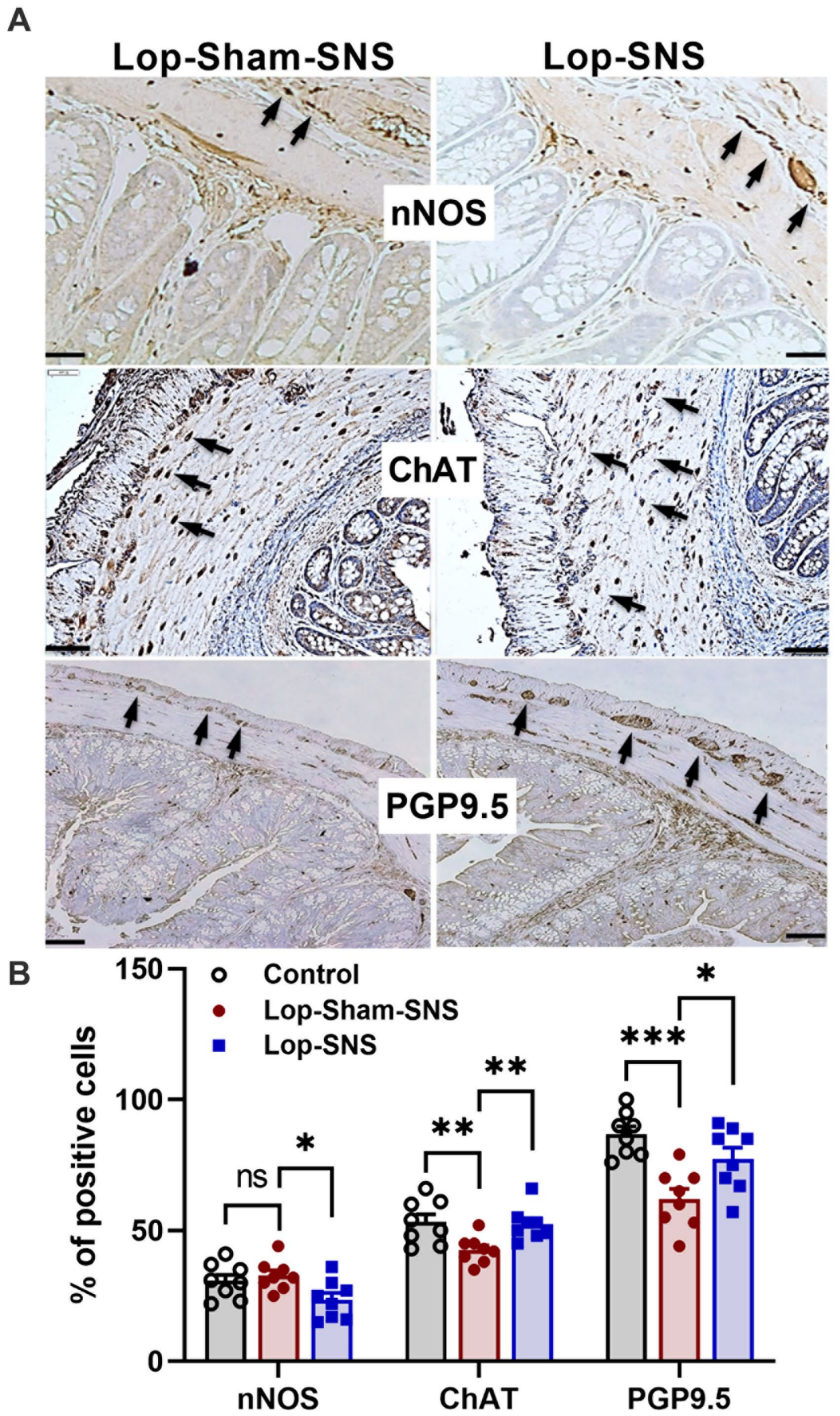
Effect of chronic SNS on enteric neurons of nNOS, ChAT and PGP9.5 of middle third of the colon. **(A)** Immunohistochemical staining of nNOS, ChAT, and PGP9.5 in sham-SNS and SNS group after 1-week treatment. Tissue with brown granular deposits (black arrow) were positive reaction and calculated per visual field in whole-mount preparations at 400×. **(B)** This graph shows the percentage of positive cells/area for nNOS, ChAT, and PGP9.5. While SNS significantly increased the number of ChAT and PGP9.5 positive cells, it was able to reduce the nNOS positive cells compared with sham-SNS. Values were represented as the means ± SE (*n* = 8). Comparisons between control and sham as well as between sham and SNS were conducted with unpaired *t*-tests (*p ≤ 0.05, **p ≤ 0.005, ***p ≤ 0.0005, ns, not significant). SNS, sacral nerve stimulation.

While Loperamide increased the protein expression of nNOS in the colon tissues of sham-SNS, SNS was able to normalize the expressions of this proteins compared to sham-SNS (*p* = 0.02; Figures 5A,B). Conversely, the ChAT protein expression that was reduced in sham-SNS treated group, was significantly amplified by SNS treatment (*p* = 0.005) (Figures 5A,B). On the other hand, as shown in Figure 6, IHC assessment of colon sections for nNOS, ChAT, and PGP9.5 showed that SNS could markedly increase the number of ChAT and PGP9.5 positive cells in muscularis propria compared to sham-SNS (*p* = 0.003 and *p* = 0.01, respectively) (Figures 6A,B). Conversely, SNS was able to reduce the protein expression of nNOS compared to sham-SNS (*p* = 0.01; Figures 6A,B).

**Figure 7:**
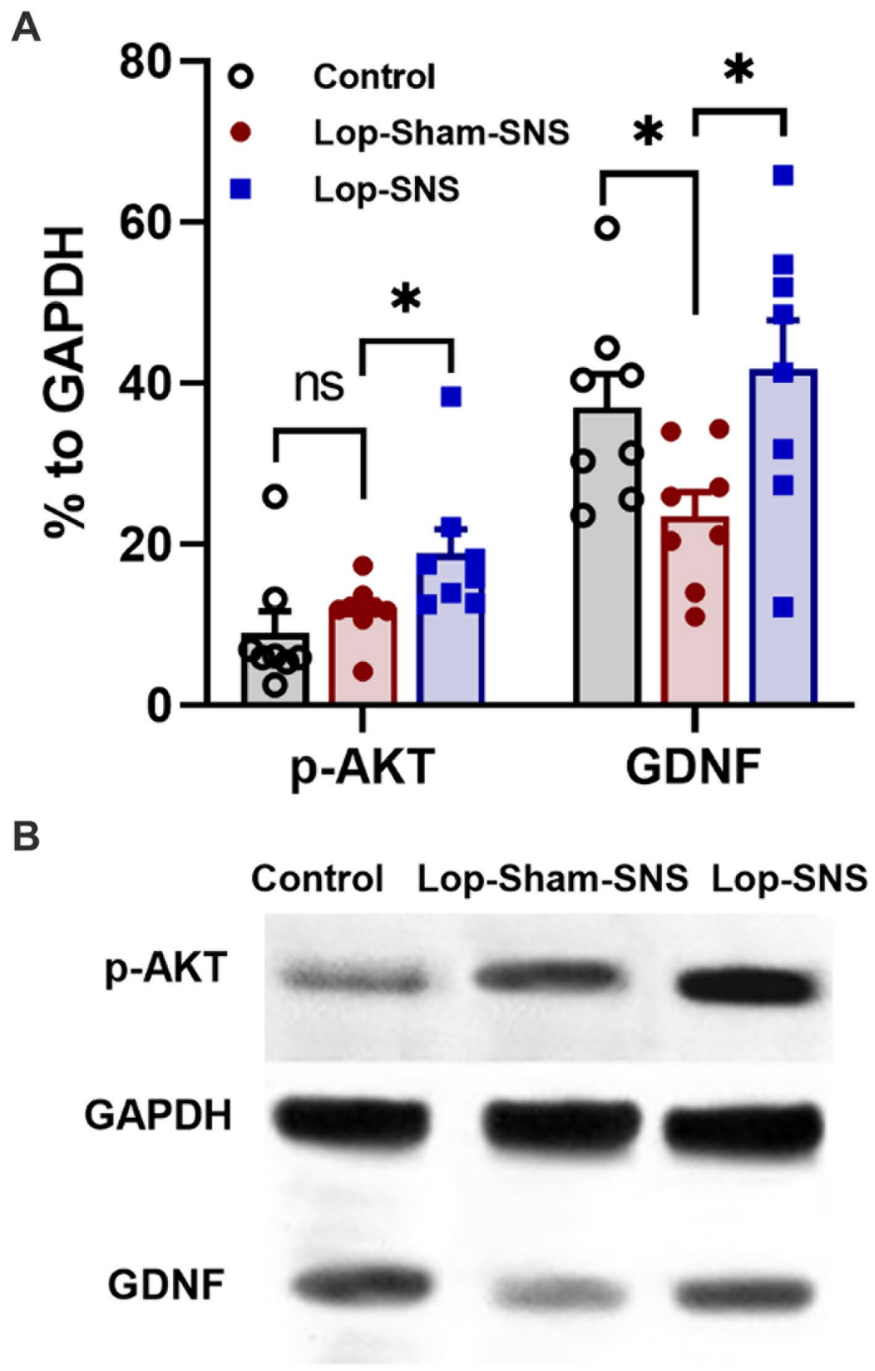
Effect of chronic SNS on protein expression of enteric neurons of p-AKT and GDNF in Loperamide-induced constipated rats in comparison with control (normal) rats. **(A)** These graphs show the analyzed protein expression of p-AKT and GDNF in colon tissue (middle third) in control, untreated (sham-SNS) and treated (SNS) groups. Loperamide treatment significantly decreased the expression of GDNF, while SNS markedly upregulated GDNF protein expression in colon tissue after 7 days compared with sham-SNS. In contrast, p-AKT that was upregulated in sham-SNS group was intensely upregulated by SNS. **(B)** Shows the protein expression of p-AKT (60 kDa), GAPDH (36 kDa), and GDNF (24 kDa) in colon tissue measured by western-blot, which has the same trends with the above graph. Values were represented as the means ± SE (*n* = 8) as percentage of relative intensity to internal control (GAPDH). Comparisons between control and sham-SNS as well as between sham-SNS and SNS were conducted with unpaired *t*-tests. SNS, sacral nerve stimulation (*p ≤ 0.05, ns, not significant). SNS, sacral nerve stimulation.

Loperamide increased the protein expressions of GDNF (*p* = 0.01) but not the protein expressions of p-AKT in the colon tissues in sham-SNS group compared to the normal rats (Figures 7A,B). Interestingly, SNS was able to normalize these protein expressions comparable to the normal rats (SNS vs. sham-SNS; p-AKT: *p* = 0.04, GDNF: *p* = 0.01, respectively).

## 5. Discussion

The current study was designed to investigate the mode of actions of SNS in improving constipation in animal model of OIC. Our major findings were (1) Acute SNS with appropriate parameters accelerated colonic transit in normal rats; (2) chronic SNS reduced WGTT in animal model of OIC by modulating the ENS as: (a) SNS increased ChAT and reduced nNOS in colon tissues, (b) SNS modulated GDNF and p-AKT to protect ENS; (3) chronic SNS improved constipation reflected as increased pellet number, wet weight and water content of feces; (4) chronic SNS balanced the sympathovagal ratio that was impaired by the treatment of Loperamide, assessed by the spectral analysis of HRV.

Several drugs, such as morphine and Loperamide have been extensively used to induce OIC in laboratory animals. Loperamide is widely accepted to induce constipation by impairing motility in the intestine and extending the stool evacuation time (Yin et al., 2018; Kim et al., 2021; Makizaki et al., 2021). In this study, we used Loperamide to induce OIC in rats and observed the human-like symptoms of constipation.

SNS is an FDA approved treatment option for urinary voiding dysfunction (Noblett and Cadish, 2014) and fecal incontinence (Benson-Cooper et al., 2013; Thin et al., 2013). Although a large number of studies have investigated the therapeutic effects of SNS for constipation, their results have not been consistent (Kenefick et al., 2002; Naldini et al., 2010; van Wunnik et al., 2012; Graf et al., 2015; Maeda et al., 2017). There were several flaws in these studies such as being-underpowered or not having well-designed (Kenefick et al., 2002; Maeda et al., 2017). More importantly, lack of standardized protocol for stimulation parameter, might have been another deficiency in these studies. Recently, our laboratory explored the most appropriate parameters of SNS for treating constipation in rat model of Loperamide-induced constipation. Huang et al. (2019), examined the effects of SNS with different stimulation frequencies (5, 15, and 30 Hz) and pulse widths (100, 210, and 500 μs) on rectal compliance in normal rats, and they found that the setting of 5 Hz and 100 μs (P1) was more potent in enhancing rectal compliance and effective in treating constipation in rats. In the current study, we compared this set of parameters with another set of parameters (P2) that was found effective in suppressing intestinal inflammation (Guo et al., 2019; Tu et al., 2020a). We studied the whole colon transit instead of only distal colon transit as done in the previous study (Huang et al., 2019), we assessed the colon transit time in whole colon by implanting a catheter at the ileocolonic junction, we observed that SNS with only P1 but not P2 was effective in accelerating whole colon transit in normal rats.

The fecal characteristics assessed in this study included feces numbers, weight and water contents (Yin et al., 2018) that are considered to be important factors for the evaluation of constipation symptoms and the therapeutic effects of SNS. In this study, although the body weight was not altered between the experimental groups (data no shown), fecal parameters (feces number, weight, and water contents) were shown to be significantly decreased following Loperamide administration. Thus, OIC was successfully established by Loperamide administration. Our observation showed that fecal parameters’ alterations were significantly improved by SNS as once daily SNS increased fecal numbers, weight, and water contents of feces, as expected.

Interestingly, our findings also showed improvement in WGTT following 1-week SNS treatment, which was consistent with the previous study (Huang et al., 2019). WGTT comprising whole gut transit including gastric emptying, intestinal and colonic transit. In our recent studies, we have shown that there is a possible sacral afferent-vagal efferent pathway that can transmit SNS stimulation to the colon tissue. We showed that SNS could be transmitted not only by local splanchnic nerves, but also via the spinal afferent to the brain stem and then by the vagal efferent to the target organ including colon (Tu et al., 2020a). This finding was supported by another study that showed SNS could increase the gastric accommodation in rats (Ye et al., 2020). In this study we have not shown the effects of SNS on gastric emptying and small intestinal transit, consist with the previous study (Huang et al., 2019). However, we might be able to presuppose that SNS might increase the motility of whole GI based on the WGTT findings. Remarkably, our autonomic function mechanistic study could support our assumption that SNS was able to enhance vagal activity and reduced the sympathovagal ratio, assessed by spectral analysis of HRV. However on the contrary, some studies showed that SNS could not alter upper GI motility in rats (Huang et al., 2019) or in patients with fecal incontinence (Damgaard et al., 2011; Worsøe et al., 2012). The difference could be attributed to different stimulation parameters and treatment regimens.

As we know, the pathological mechanisms of OIC includes the binding of exogenous opioids to peripheral μ- and δ-opioid receptors in the submucosal and myenteric plexuses of the ENS (Bagnol et al., 1997; Nelson and Camilleri, 2016) which in return might cause membrane hyperpolarization, decrease neurotransmitter release and hence, impair motility (Diego et al., 2011; Galligan and Akbarali, 2014). This has also been accompanied by an alteration in ENS neural population/functions that leads to impaired motility, as well (Diego et al., 2011; Camilleri et al., 2014). In this study, we observed that there was an ENS alteration in the colon tissue after Loperamide administration. As we know, ENS comprises excitatory and inhibitory neurons which control and coordinate the motility of the GI tract. Among these, cholinergic and nitrergic neurons expressing ChAT and nNOS, respectively, are the major excitatory and inhibitory neurons. Thus, ChAT and nNOS can be used as biomarkers to characterize the motility condition of the myenteric plexus (Sarna, 2010). On the other hand, PGP9.5 (protein gene product 9.5, or UCHL1) is a ubiquitin hydrolase which is extensively expressed in the neuronal tissues. PGP9.5 can be used for the characterization of all neurons in ENS, regardless of their actions (Akishima-Fukasawa et al., 2010). Thus, in this study we measured these cells/proteins using IHC and Western-blot analysis. Previous studies have shown that PGP9.5 cells were significantly decreased in the patients with slow transit constipation in inner circular layer (Geramizadeh et al., 2009). Consistent with this, our findings revealed that the number of PGP9.5 positive cells markedly reduced after Loperamide administration in the group that received sham-SNS treatment. Interestingly, SNS treatment effectively increased the number of PGP9.5 positive cells showing a possible neuroprotective effect of SNS on ENS neurons. Most of the neurotransmitters secreted by the ENS are identical to those found in the CNS. In general, neurons that secrete acetylcholine are excitatory, which stimulate smooth muscle contraction, increase intestinal secretions, release enteric hormones, and dilate blood vessels. ChAT is a main enzyme for acetylcholine synthesis. Our results showed that in Loperamide induced constipation, the expression of ChAT protein as well as the number of ChAT positive cells in muscularis propria of colon tissue were diminished. However, chronic SNS was able to increase the ChAT expression/ChAT positive cells of OIC rats. On the other hand, Nitric oxide (NO), as an inhibitory neurotransmitter, elicited hyperpolarization and relaxation of GI muscles (Grider et al., 1992; Foxx-Orenstein and Grider, 1996) and was necessary for relaxation of sphincter and muscle during propulsive activity in colon (Onaga et al., 2000). Previous literatures have suggested that nNOS plays an important role in maintaining the balance of intestinal contraction and relaxation, which contribute in propulsion of intestinal contents (Liu and Chen, 2006). So, it is a reasonable explanation that dysfunction of nNOS expressing neurons exist in OIC in rats. Our result revealed an increase in expression of nNOS protein, but not the cell number, in the colon tissue. Interestingly, SNS for 1-week could normalize the expression of nNOS in colon tissue.

These findings suggested that while delayed colonic transit might be associated with impairment of ChAT and nNOS in the ENS, SNS might accelerate the colonic propulsion via modulating the expression of the ChAT and nNOS. On the other hand, increasing ChAT expression might ignite this idea that SNS could ameliorate “cholinergic regulation” of colon to change the ion transport and increase the mucus secretion (Browning et al., 1977; Kim et al., 2019). As we have shown in previous study, SNS could increase the ACh release in animal model of IBD (Tu et al., 2020a). As we know, the balance between absorptive and secretory processes can rapidly change either in response to normal physiological stimuli or as a consequence of disease (Keely, 2011). ACh is the major regulator of the intestinal water transport and upon activation, it stimulates secretion from the crypts and inhibit absorption across the surface cells (Keely, 2011; Kim et al., 2019). Interestingly, our findings showed an increase in water content and wet weigh of feces after SNS treatment and thus support that SNS could affect the absorption/secretion parameters via “cholinergic regulation,” as well.

The neuropathy in the ENS damages not only neurons but supportive cells including glia. GDNF is one of the critical neurotrophic factors for the ENS (Sánchez et al., 1996). GDNF regulates many critical aspects of the ENS such as proliferation, maturation, migration and survival of the enteric neurons (Du et al., 2009). Moreover, GDNF is not only essential for formation of the ENS, but its availability also determines the total number of enteric neurons in both colon and small bowel. In addition, GDNF influences the enteric neuronal function (e.g., transmitter release, neuronal excitability; Gianino et al., 2003; Srinivasan et al., 2005; Rodrigues et al., 2011; Yin et al., 2018). Consistent with previous study (Yin et al., 2018), our findings confirmed that Loperamide could trigger downregulation of GDNF in the colon tissue. The SNS treatment significantly increased the GDNF expression in the colon tissue supporting this idea that SNS could ameliorate ENS function by increasing neuronal survival and promoting their functions.

GDNF was also reported to activate both MAP kinase (MAPK) pathway and phosphoinositide 3-kinase (PI3K) pathway (Gianino et al., 2003; Rodrigues et al., 2011). PI3K pathway was shown to promote enteric neuronal survival (Srinivasan et al., 2005). Activation of the PI3K pathway results in phosphorylation of Akt (p-AKT), which regulates a variety of cellular processes including survival, proliferation, protein translation and metabolism (Manning and Cantley, 2007). Reportedly, p-AKT is involved in reducing endoplasmic reticulum stress (ER) in various pathological condition and preventing apoptosis, as well (Mohammad-Gharibani et al., 2014; Gharibani et al., 2015). Little attention has been paid to the possible effect of GDNF and its downstream signaling pathway PI3K/Akt on OIC enteric neuronal damage. In our experiment, the activated form of AKT (p-AKT) showed a dramatic up-regulation in the colon of both sham-SNS and SNS treated groups in comparison to the normal group. This up-regulation in sham-SNS could be an indigenous reaction to Loperamide administration for promoting neuroprotection in tissue. However, SNS could increase p-AKT expression more than 25% compared to sham-SNS which can delineate its effects on ENS neuronal survival as we showed more PGP9.5 positive cells in SNS treated rats.

There were a few limitations in this study such as using only male rodents to prevent a possible compounding effect of female hormones. Hence, further studies are needed to investigate if SNS can ameliorate the Loperamide induced constipation in female rodents. Nevertheless, our data showed that the imbalance between excitatory (ChAT) and inhibitory (nNOS) neurons in the colon ENS might be one of the causes for impaired motility in opioid-induced constipation in male rats. SNS with appropriate parameters reverses the effects of Loperamide and protected the neuron loss in ENS as well as increasing the ratio of excitatory to inhibitory neurons to improve colonic transit time. By improving vagal activity, SNS could ameliorate constipation through “cholinergic regulation” of colon tissues in addition to having neuroprotective effects on ENS via GDNF-PI3K/Akt pathway. Altogether, this study suggests a great potential of the SNS for the treatment of constipation. The result of Loperamide induced constipation is sharing the abnormalities of hypomotility and reduced vagal activity with chronic functional constipation. Thus, we believe this approach as a neuromodulatory treatment can be applicable for constipated patients.

## Data availability statement

The original contributions presented in the study are included in the article/supplementary material, further inquiries can be directed to the corresponding author.

## Ethics statement

The animal study was reviewed and approved by The First Affiliated Hospital of Shandong First Medical University, Jinan, Shandong.

## Author contributions

LW: funding support of the study, study concept and design, and performing the experiment 1 and partial of experiment 2, analysis and interpretation of data, critical revision of the manuscript. PG: study design, performing the research (part of experiment 2, western-blot, and IHC), acquisition of data, analysis and interpretation of data, and drafting of the manuscript. YY: study design and help performing the experiment 1 and 2. YG: statistical analysis and interpretation of data. JY: study design and experimental guidelines. All authors contributed to the article and approved the submitted version.

## Funding

This work was mainly sponsored by The Key Research and Development Project of Shandong Province, China (NO. 2019GSF108132).

## Conflict of interest

The authors declare that the research was conducted in the absence of any commercial or financial relationships that could be construed as a potential conflict of interest.

## Publisher’s note

All claims expressed in this article are solely those of the authors and do not necessarily represent those of their affiliated organizations, or those of the publisher, the editors and the reviewers. Any product that may be evaluated in this article, or claim that may be made by its manufacturer, is not guaranteed or endorsed by the publisher.
